# Resection of Two Meters of the Small Intestine, with Recovery

**Published:** 1881-04

**Authors:** 


RESECTION OF TWO METERS OF THE SMALL INTESTINE, WITH RECOVERY. (Reported before the Academie de Médecine, January 25, 1881, by Dr. Koeberlé of Strasbourg.)

Patient, Miss K., age twenty-two, never had any disease nor indispositions, except colics, of which she has suffered at different times for the last two or three years. These had returned more severe and more frequent last year, and in October (1880), severe symptoms of intestinal strangulation recurred twice, fifteen days apart. These were relieved in both circumstances by clysters. Ever since, she suffered with continuous and intense colics, which gave her no rest, day or night, and which could hardly be subdued by hypodermics of morphia. Three points of intestinal obstruction could be inferred from the successive colics, but a positive diagnosis of the lesions could not be made.

I performed gastrotomy, November 27. There were four narrowings, more and more marked, the last being hardly .004 m. The first two were .14 m. distant. These strictures involved two meters of the small intestine, all of which were amputated between two ligatures at each end, and twelve ligatures were applied to the mesenteric vessels. The ligatures from the two free ends of the intestine were tied together in such a manner as to lay the sides opposite to the mesentery in opposition, the most favorable condition for enterectomy, and they were attached to the fibrous tissue of the *linea alba* through a suture which retained them in contact with the peritoneum at the inferior angle of the incision. The ligatures of the mesentery were brought out at the superior angle of the abdominal incision, where they were retained, together with the ligatures of the intestine, in a fixed position. The superior part of the wound was partially closed. Enterotomy was made the third day. The ligatures and sloughs came off from the twelfth to the fifteenth day. First alvine discharge took place after twenty days. On the twenty-fifth day the communication with the intestine was almost closed, and six weeks after the operation, the external wound was also closed and healed. The patient is feeling quite well, and experiences no digestive disturbance.

The temperature rose above 38° C. once on the third day. The operation lasted more than three hours, and was not made antiseptically. The peritoneal cavity was simply cleaned by towels which imbibed the serum as low down as the pelvic cavity. Patient was fed, after the second day, with solid substantial aliments (bread, meat and eggs), with an amount of liquid strictly sufficient to insure digestion. Drink was administered by rectal injections. She took thus, in the space of twenty days, seventy injections of pure water which were all retained.

*Conclusions from this and similar operations :*

1. Resection of the small intestine can be made for a considerably long portion, two, and even more, meters, without disturbing the digestive system in any appreciable manner.

2. Made under favorable conditions, this may be considered a perfectly appropriate operation.

3. Resection can be made : 1. Either by a direct operation, sewing the two ends together, and immediately closing the external wound ; 2. Or by making an incomplete apposition by sutures of the ends of the intestine, combined with an artificial anus. The second and the third are the safest procedures.

4. Resection of fibrous or cicatricial strictures, which are probably more common than we are aware, is likely to be followed by a permanent recovery. So also the resection of epitheliomas. On the contrary, resection for cancerous obstructions gives but a temporary relief, more or less marked, on account of the return of the affection, its metastasis, and the progressive degenerations of the lymphatics.

5. Keeping the intestine closed after the operation, as I did in this case, exempts the patient from any alvine discharge for many days, until the adhesions are sufficiently strong. On the other hand, the abdomen remains a little distended, thus preventing the entrance of air or of septic liquids into the peritoneal cavity. Feeding the patient solid food reduces the fæces to their minimum, and leaves no depressing result.

6. Clysters relieve the thirst, diminish the fluids in the small intestine, and free the patient from much annoyance.

